# Oncogenic Stress Induced by Acute Hyper-Activation of Bcr-Abl Leads to Cell Death upon Induction of Excessive Aerobic Glycolysis

**DOI:** 10.1371/journal.pone.0025139

**Published:** 2011-09-20

**Authors:** Michael A. Dengler, Annette M. Staiger, Matthias Gutekunst, Ute Hofmann, Malgorzata Doszczak, Peter Scheurich, Matthias Schwab, Walter E. Aulitzky, Heiko van der Kuip

**Affiliations:** 1 Dr. Margarete Fischer-Bosch Institute of Clinical Pharmacology and University of Tuebingen, Stuttgart, Germany; 2 Institute of Cell Biology and Immunology, University of Stuttgart, Stuttgart, Germany; 3 Institute of Experimental and Clinical Pharmacology and Toxicology, University Hospital Tuebingen, Tuebingen, Germany; 4 2nd Department of Internal Medicine, Oncology and Hematology, Robert Bosch Hospital, Stuttgart, Germany; University of Hong Kong, Hong Kong

## Abstract

In response to deregulated oncogene activation, mammalian cells activate disposal programs such as programmed cell death. To investigate the mechanisms behind this oncogenic stress response we used Bcr-Abl over-expressing cells cultivated in presence of imatinib. Imatinib deprivation led to rapid induction of Bcr-Abl activity and over-stimulation of PI3K/Akt-, Ras/MAPK-, and JAK/STAT pathways. This resulted in a delayed necrosis-like cell death starting not before 48 hours after imatinib withdrawal. Cell death was preceded by enhanced glycolysis, glutaminolysis, and amino acid metabolism leading to elevated ATP and protein levels. This enhanced metabolism could be linked to induction of cell death as inhibition of glycolysis or glutaminolysis was sufficient to sustain cell viability. Therefore, these data provide first evidence that metabolic changes induced by Bcr-Abl hyper-activation are important mediators of oncogenic stress-induced cell death.

During the first 30 hours after imatinib deprivation, Bcr-Abl hyper-activation did not affect proliferation but resulted in cellular swelling, vacuolization, and induction of eIF2α phosphorylation, CHOP expression, as well as alternative splicing of *XPB*, indicating endoplasmic reticulum stress response. Cell death was dependent on p38 and RIP1 signaling, whereas classical death effectors of ER stress, namely CHOP-BIM were antagonized by concomitant up-regulation of Bcl-xL.

Screening of 1,120 compounds for their potential effects on oncogenic stress-induced cell death uncovered that corticosteroids antagonize cell death upon Bcr-Abl hyper-activation by normalizing cellular metabolism. This protective effect is further demonstrated by the finding that corticosteroids rendered lymphocytes permissive to the transforming activity of Bcr-Abl. As corticosteroids are used together with imatinib for treatment of Bcr-Abl positive acute lymphoblastic leukemia these data could have important implications for the design of combination therapy protocols.

In conclusion, excessive induction of Warburg type metabolic alterations can cause cell death. Our data indicate that these metabolic changes are major mediators of oncogenic stress induced by Bcr-Abl.

## Introduction

It is accepted that activation of growth-promoting oncogenes by either mutation, gene fusion, or amplification, is necessary but not sufficient for malignant outgrowth. In fact, tumor cells are often addicted to the activity of these oncogenes, which makes them perfect therapeutic targets [1]. However, a prerequisite for an unscheduled proliferation of tumor cells upon activation of oncogenes seems to be the simultaneous inhibition of tumor suppressor mechanisms such as over-expression of anti-apoptotic proteins or inactivation of tumor suppressors [2], [3]. This theory has been arisen from the finding that normal cells respond to hyper-activation of oncogenes by the induction of genetically encoded programs such as apoptosis or senescence [4]–[6]. Therefore, extreme activation of a growth promoting oncogene appears to disturb cellular homeostasis, a phenomenon known as oncogenic stress. Senescence or cell death pathways induced as consequences of oncogenic stress have been primarily studied in cells derived from solid tumors rather than hematopoietic malignancies, which are often triggered by constitutively active oncogenic fusion-proteins such as Bcr-Abl.

Bcr-Abl is derived from a balanced translocation between the chromosomes 9 and 22 and can be detected in almost all patients with chronic myeloid leukemia (CML) and in around 20% of cases of acute lymphoblastic leukemia (ALL). The outcomes for patients with Bcr-Abl positive leukemias have been substantially improved with the introduction of the Abl kinase inhibitor imatinib [7]. In ALL patients, however, imatinib monotherapy produces only a transient response. Combination therapy strategies using imatinib and conventional chemotherapy including corticosteroids such as prednisone and dexamethasone turned out to be superior to the single administration [8].

The constitutively active tyrosine kinase Bcr-Abl acts upstream of numerous growth and antiapopototic signaling pathways [9]. Via docking to the adaptor molecules and guanine-nucleotide exchangers GRB2 and SOS, Bcr-Abl activates the Ras-MAPK pathway [10], which in turn enhances the level of the antiapoptotic protein Bcl-2 [11]. Bcr-Abl also constitutively activates PI3K/Akt via the scaffold adapter GAB2. PI3K/Akt acts upstream of mTOR, a pivotal regulator of protein synthesis [12], and inhibits the proapoptotic BH3 only protein BAD [13]. Bcr-Abl also phosphorylates several Src kinase family members such as Hck, and Lyn [14] which is required for induction of Ph+ALL [15]. Several signaling pathways downstream of Src kinases prevent induction of apoptosis, e.g. Hck recruits STAT5 which not only modulates proliferation but also upregulation of the antiapoptotic Bcl-xL [16].

Interestingly, Bcr-Abl has also been associated with metabolic reprogramming [17], [18], a phenomenon that is a commonly accepted hallmark of malignant cells [19]. Already in 1924 Otto Warburg postulated that in contrast to most cells in normal tissue, cancer cells generate their energy by “fermentation” of glucose into lactate even when sufficient oxygen is present [20]. This phenomenon (today known as aerobic glycolysis or the Warburg effect), which facilitates uptake and incorporation of nutrients into the biomass of cancer cells allowing them to sustain higher proliferative rates [21], also holds true for Bcr-Abl transformed cells. Bcr-Abl positive cells express the high affinity GLUT-1 glucose transporter and show an increased glucose uptake [17]. When the intracellular glucose content exceeds the capacity to assimilate glucose, the cells respond with an induction of HIF-1α that is required to eliminate excess glucose carbon from these cells in the form of lactate [18]. Therefore, probably via upregulation/stabilization of HIF-1α, Bcr-Abl switches cellular metabolism to increased lactate production and reduced oxygen consumption. This altered metabolic regulation depends on Bcr-Abl kinase activity since inhibition of Bcr-Abl by imatinib reduces HIF-1α [18] and changes glucose metabolism back from aerobic glycolysis to mitochondrial citrate cycle [22].

Recently, it has been demonstrated that acute activation of Bcr-Abl in imatinib resistant, Bcr-Abl over-expressing cells can induce cell death [23]. In the present study, we investigated the correlation between acute Bcr-Abl activation, altered metabolism, and cell death induction in Bcr-Abl over-expressing cells after imatinib withdrawal. We here describe that hyper-activation of Bcr-Abl leads to a significantly enhanced rate of aerobic glycolysis and glutaminolysis. This “overshooting” metabolism is responsible for the induction of a p38 and RIP-1 dependent cell death. These data provide the first evidence that excessive induction of Warburg type metabolic changes can cause cell death.

## Results

### Effects of imatinib on Bcr-Abl over-expressing cell clones

**Figure 1:**
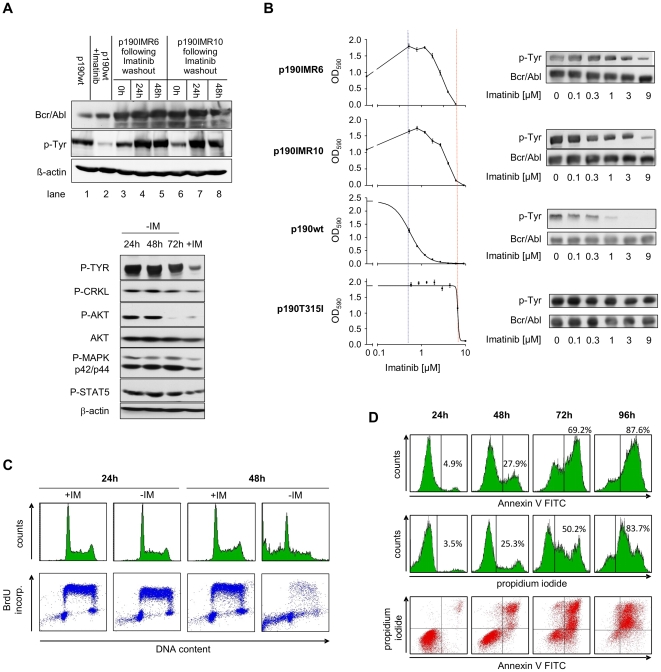
Phenotype of Bcr-Abl over-expressing imatinib resistant (IMR) cells. (A) Bcr-Abl expression and activity. Upper panel: Bcr-Abl protein level and autophosphorylation in imatinib-sensitive cells (p190wt) in comparison to imatinib-resistant cell clones (IMR6 and IMR10) in the presence or absence of 2 µM imatinib. Lower panel: activity of Bcr-Abl downstream-signaling in IMR cells in the presence of imatinib and following imatinib withdrawal. (B) Effect of imatinib on survival (as measured by MTT) of Bcr-Abl over-expressing p190IMR clones in comparison to cell expressing wtBcr-Abl (p190wt) or T315IBcr-Abl (p190T315I). IC50 values of p190wt and p190T315I cells are indicated with dotted lines (C) Effect of imatinib deprivation on cell cycle distribution. Cells were cultivated in absence or presence of imatinib for indicated time periods and pulse treated with BrdU for 45 min. Cells were then fixed and evaluated for DNA content and synthesis via BrdU and propidium iodide (PI) staining and analyzed by flow cytometry. (D) Effect of imatinib deprivation on cell viability. Cells were stained with Annexin V or PI alone (upper two panels) or with the combination of both Annexin V and PI (lower panel) and analyzed by Flow cytometry.

To investigate cellular responses following hyper-activation of Bcr-Abl oncogene we established a cell model based on Bcr-Abl over-expressing BaF3 cells selected by continuous cultivation in the presence of 2 µM imatinib. Two imatinib resistant (IMR) cell clones (termed as p190IMR6 and p190IMR10) were selected for the study. Both clones were negative for Abl kinase mutations (not shown) and showed Bcr-Abl overexpression (figure 1A, upper panel; lanes 3–8). In these clones Bcr-Abl phosphorylation in presence of imatinib was comparable to that observed in Bcr-Abl positive parental cells without imatinib (Figure 1A, upper panel; lanes 1, 3 and 6). Imatinib withdrawal leads to hyper-phosphorylation of Bcr-Abl in IMR cells (Figure 1A, upper panel; lanes 4, 5, 7, and 8) causing an excessive stimulation of Bcr-Abl downstream pathways as indicated by enhanced phosphorylation of Crkl, Akt, MAPK, and STAT5 (Figure 1A, lower panel).

Both cell lines were partially resistant to imatinib. Concentrations exceeding 4 µM rapidly killed both cell lines. Interestingly, imatinib withdrawal also led to a loss of viability of these cell lines (Figure 1B). Whereas the cell death after high dose imatinib exposure was related to loss of Bcr-Abl phosphorylation, imatinib withdrawal induced loss of cell viability was accompanied by an excessive hyper-phosphorylation of this protein.

To investigate the dependency of these cells on imatinib we performed cell cycle analysis and AnnexinV-staining. During the first 24 hours after imatinib withdrawal the cells did not show any evidence for changes in cell cycle, proliferation (Figure 1C), and cell viability (Figure 1D). First signs of cell death were apparent 48 hours after imatinib withdrawal as indicated by an increased number of sub-G1 cells (Figure 1C). To characterize the mechanism of cell death induced by Bcr-Abl hyper-activation, we performed Annexin V and propidium iodide (PI) double staining. Classical apoptosis is characterized by a lag time between Annexin positivity (phosphatidylserine exposure on the cell surface) and PI positivity (membrane permeabilization), whereas in necrosis these events coincide (Figure S1; [24]). Our data show that already at 48 h when first signs of cell death appeared as well as at later time points most dead cells were positive for PI indicating that cell death induced by imatinib withdrawal is more necrosis-like rather than classical apoptotic (Figure 1D). Cell death was not only prevented by optimal concentrations of imatinib but also by partial inhibition of Bcr-Abl-activity by other Abl inhibitors such as dasatinib and nilotinib indicating that Bcr-Abl-hyper-phosphorylation is indeed responsible for loss of viability observed in IMR cells upon imatinib withdrawal (Figure S2).

These results demonstrate that hyper-activation of Bcr-Abl achieved by imatinib withdrawal leads to a delayed induction of a necrosis-like cell death.

### Imatinib withdrawal influences cellular metabolism

**Figure 2:**
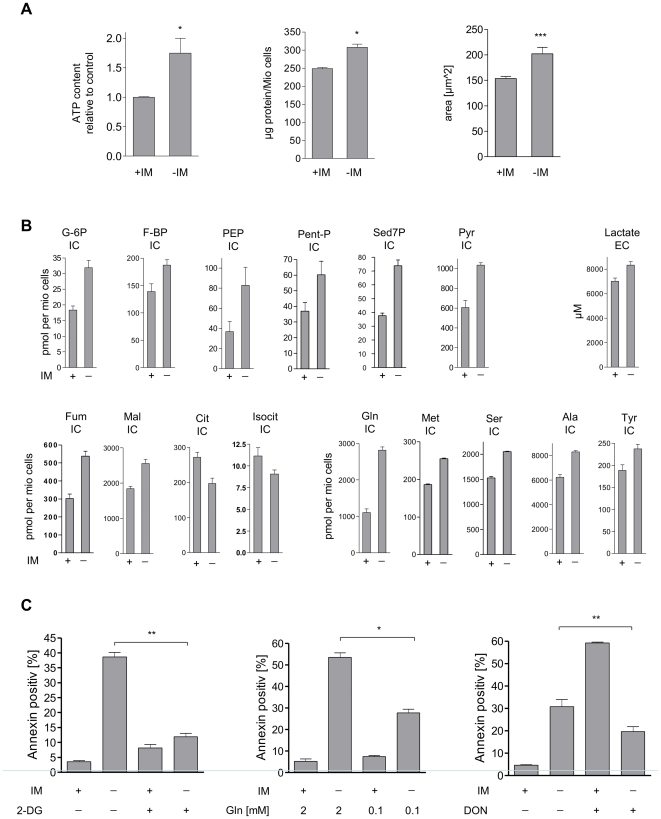
Imatinib withdrawal alters cell metabolism in IMR cells. (A) ATP content (*: *P* = 0.0313, left panel), protein content (*: *P* = 0.0367, middle panel), and cell size (***: *P*<0.0001, right panel) of IMR cells cultivated with imatinib or 24 hours after imatinib withdrawal. Values reflect means ± SD from 3 experiments. (B) Effects of imatinib withdrawal on glucose and amino acid metabolism. Cells were cultivated in presence or absence of imatinib for 24 hours and then harvested and prepared for GC-MS or LC-MS-MS. Values reflect means ± SD from 3 experiments. (C) Induction of cell death in IMR cells cultivated with imatinib or 48 hours after imatinib withdrawal in the presence or absence of 2-deoxy-D-glucose (**: *P* = 0.0048, upper panel), in medium with different glutamine concentrations (*: *P* = 0.02, middle panel) or in presence or absence of 6-diazo-5-oxo-l-norleucine (DON; **: *P* = 0.0014). After treatment cells were harvested, stained for Annexin-V-FITC and cell death was quantified by FACS analysis. Values reflect means ± SD from 3 experiments.

Although the cells did not show any changes in proliferation after 24 h of imatinib withdrawal, we could observe significant changes in intracellular ATP and protein content as well as an increase in cell size at this time point (Figure 2A). This could represent a primary metabolic reaction upon Bcr-Abl hyper-activation. It has been demonstrated that Bcr-Abl expression is associated with an increased rate of glycolysis [18], [22]. Therefore, we analyzed the metabolic profile of IMR cells 24 h after imatinib withdrawal using mass spectrometry assays for the detection of a broad variety of metabolites as described previously [25]–[27]. In fact, these cells showed an increase of glycolysis and pentose-phosphate pathway intermediates as shown for glucose-6-phosphate, fructose-1,6-bisphosphate, phosphoenolpyruvate, pentose-phosphates, seduheptulose-7-phosphate and pyruvate (Figure 2B). Together with the increased extracellular concentrations of lactate (Figure 2B) these data confirm the view that Bcr-Abl activation leads to an elevated aerobic glycolysis. Hyper-activation of Bcr-Abl also led to a consistent increase of the intracellular concentration of all proteinogenic amino acids as displayed for glutamine, methionine, serine, alanine, and tyrosine (Figure 2B). Notable was the more than twofold increase of glutamine upon imatinib withdrawal, indicating that glutamine is also used as an energy source through elevated glutaminolysis using several steps of the tricarboxylic acid cycle. This was supported by our finding that the levels of tricarboxylic acid cycle intermediates change divergently upon hyper-activation of Bcr-Abl: the intracellular concentrations of fumarate and malate were increased whereas the citrate and isocitrate levels were decreased (Figure 2B). Importantly, this enhanced cellular metabolic activity upon acute hyper-activation of Bcr-Abl was not beneficial for the cells as proposed by Warburg [28]. On the contrary, enhanced glycolysis could be linked to the cell death observed 48 hours after imatinib withdrawal as inhibition of glycolysis by 2-deoxyglucose (2-DG) completely rescued cells from imatinib withdrawal induced death (Figure 2C, left panel). A significant, although incomplete, inhibition of cell death was also observed upon partial deprivation of glutamine from the medium and inhibition of glutaminase activity using the glutaminase inhibitor 6-diazo-5-oxo-l-norleucine (DON; Figure 2C, middle and right panels). Although DON turned out to be toxic in presence of imatinib, it significantly reduced imatinib withdrawal induced cell death. Interestingly, cellular ATP levels were only slightly decreased in imatinib deprived cells treated with 2-DG or DON (Figure S3) indicating that these cells can produce ATP from either glucose or glutamine.

These experiments indicate that not only enhanced glycolysis but also enhanced glutaminolysis is involved in cell death induced by Bcr-Abl-mediated oncogenic stress.

### Imatinib withdrawal induces cellular swelling and severe ER stress

**Figure 3:**
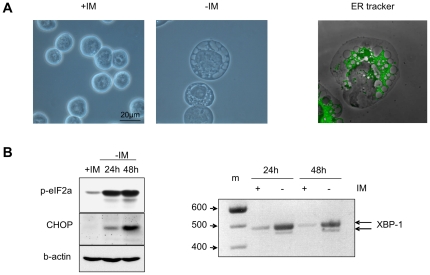
Imatinib withdrawal induces ER stress. (A) Phase contrast images of IMR cells cultivated with or without imatinib for 24 hours (left panels) and p190IMR cells stained with ER-TrackerTMGreen dye (Invitrogen) 24 hours after imatinib withdrawal (right panel). (B) Imatinib withdrawal induces prototypical ER stress markers. Left panel: phosphorylation of eIF2α and protein level of CHOP in presence of imatinib and upon imatinib withdrawal evaluated after indicated time periods by western blot analysis. Right panel: agarose gel electrophoresis of *XBP-1* splicing variants amplified by PCR.

The enhanced metabolic rate in imatinib deprived Bcr-Abl over-expressing IMR cells led to remarkable morphologic changes. Microscopically we observed not only cellular swelling but also cytoplasmic vacuolization with mostly large ballooned vacuoles (Figure 3A). Such severe cytoplasmic vacuolization may reflect endoplasmatic reticulum (ER) stress [29]. We therefore stained cells with ER tracker upon imatinib withdrawal. ER staining revealed a huge dilation of the ER cisternae indicating that the vacuoles observed upon imatinib withdrawal were formed by the ER (Figure 3A). To further confirm that Bcr-Abl hyper-activation induces ER stress we also investigated expression of typical ER stress markers. Western blot analysis revealed that imatinib withdrawal increased phosphorylation of eIF2α on serine 51 and induced the ER stress-mediated apoptotic protein CHOP (Figure 3B, left panel), both being distinct markers of ER stress [30]. Another typical ER stress protein is the transcription factor XBP-1. *XBP-1* is up-regulated and the transcript is converted into mature mRNA by unconventional splicing mechanisms upon ER stress [31]. As shown in Figure 3B (right panel), deprivation of imatinib led to induction of *XBP-1* expression and to its alternative splicing. These results demonstrate that hyper-activation of Bcr-Abl results in a strong ER stress response.

Recent findings indicate that ER stress is also a potent inductor of autophagy. We therefore next examined if inhibition of autophagy might influence cell death. In our cellular system autophagy was probably induced because Beclin-1 and ATG7 were up-regulated upon imatinib withdrawal. However, neither the autophagy inhibitor 3-Methyladenin (3-MA) nor silencing of Beclin or ATG7 (Figure S4) had any influence on induction of cell death upon imatinib withdrawal.

Therefore, our data indicate that autophagy is induced by acute Bcr-Abl activation but is not involved in the execution of the delayed cell death.

### Cell death is independent of CHOP-BIM mediated apoptosis but depends on RIP1 and p38 activation

**Figure 4:**
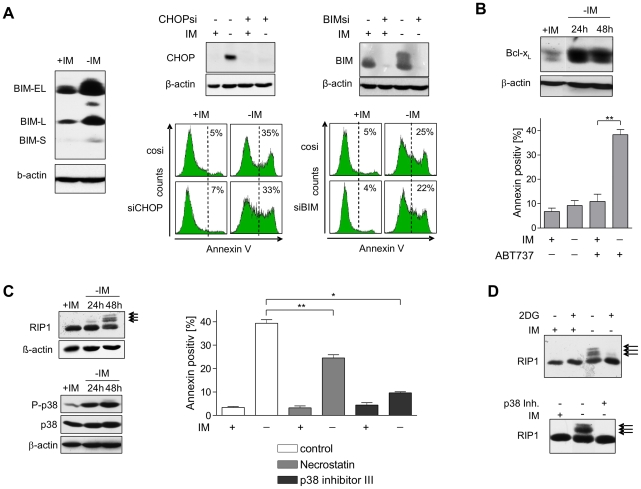
Imatinib deprivation leads to non-apoptotic cell death mediated by p38 and RIP1. (A) BIM is highly induced but does not affect cell death upon imatinib deprivation. Left panel: Cells were cultivated in presence or absence of imatinib for indicated time periods and harvested for detection of BIM protein variants in western blot analysis. Right panel: cells were transfected with control siRNA or siRNA targeting BIM or CHOP. After 16 hours medium was changed and cells were further cultivated with or without imatinib for 48 hours. Cells were then harvested for western blot analysis or quantification of cell death by Annexin staining and flow cytometry. (B) Apoptosis is antagonized by a parallel induction of Bcl-xL. Upper panel: western blot analysis of Bcl-xL from cells cultivated with or without imatinib. Lower panel: inhibition of Bcl-xL by ABT-737 leads to induction of apoptosis early after imatinib withdrawal (***: *P* = 0.0012). Cells were cultivated in the presence and absence of imatinib and ABT-737 for 24 hours and then harvested for Annexin V-FITC staining and flow cytometry. Values reflect means ± SD from 3 experiments. (C) RIP1 and p38 activity is induced upon imatinib withdrawal and responsive for induction of cell death. Left panel: cells were cultivated with or without imatinib and harvested for western blot analysis using specific antibodies recognizing p38-Thr180/Tyr182, p38 or RIP1. Right panel: inhibition of RIP1 by Necrostatin-1 (**: *P* = 0.0076) and inhibition of p38 (*: *P* = 0.0102) antagonize cell death upon imatinib withdrawal. Cells were incubated with or without Necrostatin-1 and p38 inhibitor III and imatinib for 48 hours and then harvested for Annexin V staining and flow cytometry. Values reflect means ± SD from 3 experiments. (D) Inhibition of glycolysis or p38 activity abrogates RIP1 posttranslational modification upon Imatinib withdrawal. Cells were incubated with or without 2-deoxy-D-glucose or p38 inhibitor III and Imatinib as indicated. 48 hours after Imatinib withdrawal cells were harvested for western blot analysis.

It has been demonstrated that severe ER stress induces apoptosis by activating the BH3-only Bcl-2 family member BIM via CHOP-mediated transcriptional induction [32]. Indeed, BIM-EL, BIM-L, and BIM-S were elevated upon imatinib withdrawal in Bcr-Abl overepressing cells (Figure 4A, left panel). Interestingly, however, despite an almost complete siRNA-mediated down-modulation of CHOP and BIM (Figure 4A, middle and right upper panels), neither silencing of CHOP nor BIM had any effect on induction of cell death in these cells (Figure 4A, middle and right lower panels). These results indicate that the ER stress triggered apoptotic pathway via IRE, CHOP, and BIM does not play a dominant role for induction of cell death in these cells despite its induction upon imatinib withdrawal. This was further supported by the result that inhibition of caspases by zVAD-fmk was not able to prevent but rather enhanced imatinib withdrawal induced cell death (Figure S5). It appears feasible that BIM-induced apoptosis is blocked by the antiapoptotic Bcl-2 family member Bcl-xL which is also up-regulated upon Bcr-Abl hyper-activation (Figure 4B, upper panel). This hypothesis is supported by the observation that in the presence of the BH-3 mimetic ABT-737, which is able to bind and inhibit Bcl-xL, cell death was induced already 24 hours after imatinib withdrawal (Figure 4B, lower panel). In contrast to the delayed cell death in absence of ABT-737, this early cell death was a predominant apoptotic process since approximately half of the dead cells were positive for Annexin but negative for propidium iodide (Figure S6).

Together, these results indicate that the deregulated metabolism induces severe ER stress and also apoptotic signals through the induction of the pro-apoptotic protein BIM. However, execution of apoptosis is blocked by the concomitant induction of Bcl-xL at early time points after imatinib withdrawal.

It is known that inhibition of apoptosis by overexpression of antiapoptotic Bcl-2 proteins can result in induction of RIP1-dependent programmed necrosis [33]. RIP1 is a death domain containing protein kinase that complexes with TRAF2 to activate MEKK4 and ASK1. Both MEKK4 and ASK1 activate p38 MAPKs via MKK3 and MKK6 [34]. As shown in Figure 4C (left upper panel), RIP1 activity was induced upon imatinib deprivation as demonstrated by the occurrence of additional slower migrating RIP1 signals, indicative for RIP1 autophosphorylation [35]. An enhanced phosphorylation was also observed for p38 upon imatinib deprivation (Figure 4C, left lower panel). Inhibition of RIP1 by Necrostatin-1 and even more effectively p38-MAPK inhibition by the p38 inhibitor III rescued cells from imatinib deprivation induced cell death (Figure 4C, right panel), indicating that these proteins play a major role for cell death upon oncogenic stress. RIP1 activation was completely blocked upon inhibition of aerobic glycolysis by 2DG further supporting that this pathway is indeed activated by the overshooting metabolism upon acute Bcr-Abl activation (Figure 4D). Interestingly, inhibition of p38 also blocked RIP1 activation indicating the existence of a positive feedback loop (Figure 4D).

### Corticosteroids prevent imatinib deprivation induced cell death

**Figure 5:**
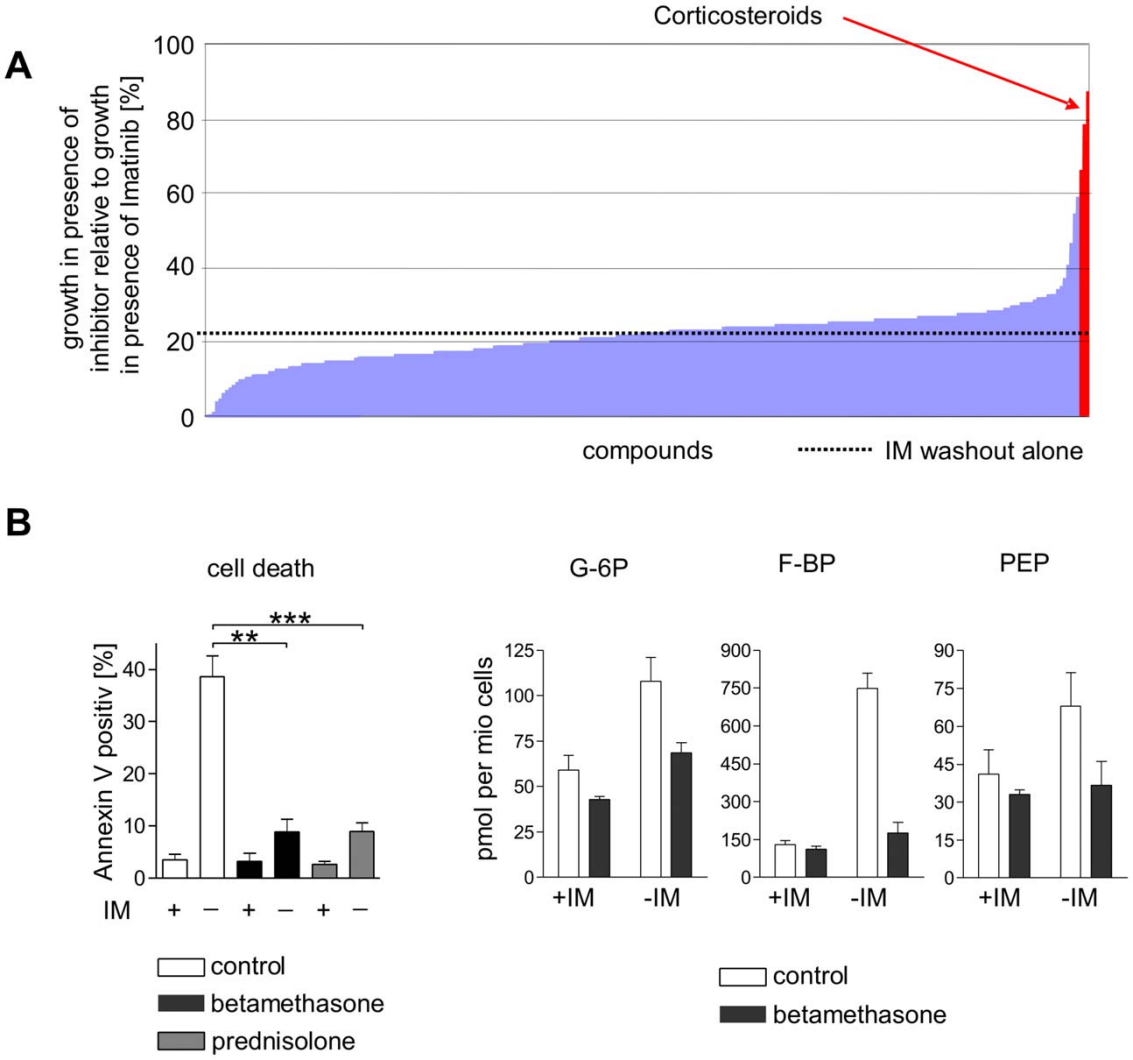
Modulation of cell death and altered metabolism induced by imatinib withdrawal in IMR cells. (A) Effects of a panel of 1,120 approved small molecule inhibitors (Prestwick library) on cell survival upon imatinib-deprivation. Cells were incubated with DMSO control, inhibitors and with or without imatinib for 48 hours and cell survival was then measured by MTT colorimetric analysis. Values reflect percent survival of cells treated with inhibitor in absence of imatinib in relation to imatinib treated controls. Survival of imatinib withdrawal alone is indicated by a dotted line. (B) Effects of betamethasone (**: *P* = 0.001) and prednisolone (***: *P*<0.0001) on induction of cell death (left panel) and glucose metabolism (right panel) on imatinib withdrawal induced cell death. Cells were cultivated in the presence or absence of betamethasone, prednisolone, and imatinib for 48 hours and then harvested for cell death quantification by Annexin V staining and flow cytometry (left panel) or for fixation and preparation for GC-MS or LC-MS-MS (right panel). Values reflect means ± SD from 3 experiments.

Next, we used a high throughput screening assay to determine whether approved drugs would interfere with imatinib withdrawal induced cell death. For this, we screened the Prestwick Chemical Library® which contains 1,120 marketed drugs by means of MTT assay. Upon imatinib deprivation cell viability was reduced to 20.7% of imatinib-treated control cells in the absence of inhibitors (DMSO controls; Figure 5A, indicated with blue line). Among those 1,120 compounds, only 17 were identified capable to significantly protect Bcr-Abl over-expressing cells from imatinib withdrawal induced cell death resulting in a survival rate of more than 60% (Figure 5A, red bars). Interestingly, 16 of those turned out to be corticosteroids such as the glucocorticoids betamethasone and prednisolone. To confirm these hits of our screening results, we determined the percentage of cell death upon imatinib withdrawal in presence or absence of betamethasone and prednisolone by Annexin-V staining. As shown in Figure 5B (left panel), both compounds almost completely rescued Bcr-Abl hyper-activated cells from imatinib deprivation induced cell death. Importantly, treatment with corticosteroids was sufficient to normalize glucose metabolism: in Bcr-Abl hyper-activated cells betamethasone reduced key intermediates of glycolysis, such as glucose-6-phosphate, fructose-1,6-bisphosphate, and phosphoenolpyruvate, to levels comparable to those observed in imatinib treated controls (Figure 5B, right panel). These results support our observation that cell death upon Bcr-Abl overactivation is mediated by enhanced glucose and glutamine metabolism which can be antagonized by corticosteroids.

### Corticosteroid treatment enhances Bcr-Abl transformation and selects for Bcr-Abl overexpression

**Figure 6:**
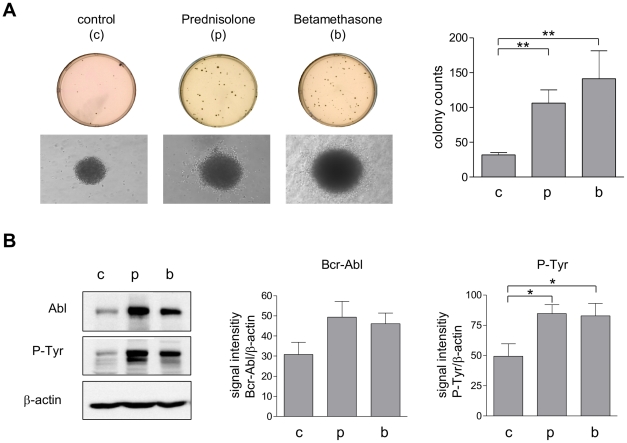
Corticosteroids render the BaF3 cells permissive for the transforming activity of Bcr-Abl. (A) Colony number and colony size of BaF3 cells transfected with an expression vector containing e1a2*bcr-abl* as insert in presence or absence of prednisolone or betamethasone. 48 hours after transfection cells were counted and further cultivated in semisolid medium with or without prednisolone or bethamethasone. Left panel: photographs of representative wells (from 6-well plates). Right panel: colony numbers of cells transfected in absence (c) or presence of prednisolone (p; **: *P* = 0.0013) or betamethasone (b; **: *P* = 0.0013). Values reflect means ± SEM of triplicates from 3 independent experiments (B) Bcr-Abl expression and autophosphorylation of *bcr-abl* transfected cell clones. Left panel: representative example. Right panels: relative Bcr-Abl protein levels in absence (c) or presence of prednisolone (p) or betamethasone (b) and relative autophosphorylation in absence (c) or presence of prednisolone (p; *: *P* = 0.0126) or betamethasone (b; *: *P* = 0.0364) were measured by forming the ratio of the densitometric values of bands pooled from results of 10 clones.

Our results so far demonstrate that hyper-activation of the oncogene Bcr-Abl led to induction of cell death as a consequence of an enhanced metabolic activity. To study whether this cell death limits the transforming activity of Bcr-Abl, we transfected BaF3 cells with an expression vector containing e1a2*Bcr-Abl* as insert in the presence or absence of prednisolone or betamethasone. As shown in Figure 6A, transfection efficacy was significantly enhanced in the presence of corticosteroids despite of a markedly reduced proliferation capacity of more than 50% in BaF3 cells (not shown). Furthermore, the proliferation rate of Bcr-Abl transfected cells was enhanced in presence of corticosteroids as indicated by an elevated size of the colonies (Figure 6A, left panel). Interestingly, most of the cell clones cultivated in presence of corticosteroids were characterized by higher expression and activation of Bcr-Abl (Figure 6B). Together, these data indicate that corticosteroids enhance cell transformation through Bcr-Abl and select for Bcr-Abl overexpression.

## Discussion

Oncogenes are altered version of normal genes (proto-oncogenes) with deregulated activity [36]. Although they are best known for their role in induction of cell proliferation and inhibition of apoptosis, oncogenes can also, under certain conditions, initiate cellular disposal programs. For example, expression of oncogenic Ras in normal cells is sufficient to trigger cell death [37]. Here we show that the same principle holds true for another oncogene, namely Bcr-Abl. In Bcr-Abl over-expressing cells partially resistant to imatinib, withdrawal of this TKI leads to a protracted induction of cell death. Our data indicate that this lethal oncogenic stress response is caused by an enhanced aerobic glycolysis and glutaminolysis.

According to our knowledge, these data provide the first evidence linking enhanced aerobic glycolysis and glutaminolysis to induction of cell death. High rates of glucose utilization and lactate production despite the presence of sufficient oxygen are the most common metabolic hallmarks of malignant cells [38]. This metabolic switch from mitochondrial glucose oxidation to aerobic glycolysis, also known as Warburg effect, is an efficient strategy of tumor cells for both energy production and maximizing their anabolic growth. In addition, the accompanying repression of mitochondrial respiration seems to serve as a protective mechanism for tumor cells to avoid excessive production of ROS [39]. Our data indicate that an excessive glycolysis upon acute hyper-activation of oncogene-dependent pro-proliferative and antiapoptotic signals can have opposite effects and induces cell death. The paradoxical observation that Bcr-Abl mediated excessive glucose consumption can suppress leukemic cell growth has also been previously reported [18]. Zhao et al. investigated HIF-1α induced effects on metabolism in Bcr-Abl over-expressing clones. They found that these cells display an increase in glycolysis but a concomitant reduction in cell counts. These authors interpreted these data as evidence for diminished cell proliferation [18]. Our data show that this reduced cell number is more likely the result of induction of cell death. We further demonstrate that inhibition of glycolysis or glutaminolysis is sufficient to abrogate cell death upon hyper-activation of Bcr-Abl, strongly supporting the hypothesis that cell death is indeed mediated by an enhanced glucose metabolism. Numerous experiments have demonstrated that tumor cells strongly rely on aerobic glycolysis [38]. Our experiments show that induction of aerobic glycolysis above a critical level can be lethal for transformed cells. Furthermore, they demonstrate that abnormal activation of cellular oncogenes is capable of inducing glycolysis above this lethal threshold. The biological significance of this observation is further supported by the fact that compounds antagonizing these metabolic alterations render the cells permissive for the transforming activity of Bcr-Abl.

Induction of cellular death upon Bcr-Abl hyper-activation is a remarkably slow process. During the first 24 hours after imatinib withdrawal we observed neither signs of cell death nor any changes in cell cycle distribution. During this time period the cells showed an enhanced anabolic metabolism paralleled by an increase in PI3K, Ras, and STAT signaling. Moreover, the cells displayed both morphological and molecular changes indicative for a prototypical ER stress response. Interestingly, a comparable cellular response has also been described in primary melanocytes following exogenous expression of another oncogene, namely the oncogenic form of HRAS [40] supporting the hypothesis that ER stress might represent a more general limiting factor for the transforming capacity of oncogenes.

ER stress can be activated by several signals such as the unfolded protein response, changes in protein and lipid metabolism [41] and alterations of the redox or metabolic state of a cell [42], [43]. Dependent on cell type and the given status of a cell, severe ER stress can have different consequences such as the induction of apoptosis [44] or the initiation of autophagy [45]. At early time points after Bcr-Abl hyper-activation both apoptosis and autophagy related proteins were up-regulated, indicating that different cell death mechanisms are activated. However, cell death development induced either by apoptotic or autophagic signals was antagonized by a parallel induction of the antiapoptotic Bcl-xL protein. If execution of apoptosis is blocked by high levels of antiapoptotic proteins or by defects in activation and/or function of caspases, alternative non-apoptotic cell death pathways can be activated, such as RIP1-dependent programmed necrosis [33]. RIP1 is a death domain containing protein kinase that complexes with TRAF2 to activate MEKK4 and ASK1. Both MEKK4 and ASK1 then activate p38 via MKK3 and MKK6 [34]. Indeed, our data show that both RIP1 as well as p38 were activated upon Bcr-Abl hyper-activation. Inhibition of RIP1 by Necrostatin-1 partially rescued cells from imatinib deprivation-induced cell death, indicating that this necrotic/necroptotic signaling pathway substitutes for the blocked apoptosis at least partially. This is not only supported by our observation that after Bcr-Abl hyper-activation most dead cells showed typical features for necrosis in Annexin V and propidium iodide staining (Figure 1D) but also by the fact that zVAD-fmk enhances, rather than blocks cell death development (Figure S4; [46]). Importantly, inhibition of aerobic glycolysis by 2DG blocked activation of RIP1, indicating that this kinase activity dependent pathway is indeed activated by the overshooting metabolism upon hyper-activation of Bcr-Abl. Interestingly, inhibition of p38 was even more effective in rescuing cells from Imatinib deprivation induced death. It has been demonstrated that p38 inhibition may reduce HIF-1α protein expression and therefore negatively regulate aerobic glycolysis [47]. The HIF-1α induced changes in glycolysis in Bcr-Abl over-expressing cells [18] may therefore be dependent on p38 activation. This hypothesis is supported by our finding that inhibition of p38 had the same effect on RIP1 activity as inhibition of glycolysis. Therefore, p38 may also act upstream of RIP1 via induction of glycolysis.

In conclusion our data provide insight into the molecular mechanisms connecting oncogene-altered metabolism with cell death. The above observations support the hypothesis that overshooting glycolysis and glutaminolysis upon acute Bcr-Abl hyper-activation trigger a RIP1 and p38 dependent necrosis-like cell death. This ability to induce cell death by hyper-activation of Bcr-Abl may also be relevant to other oncogenes and their pathways such as the PI3K pathway which regulates many of the normal metabolic consequences of growth factor stimulation [38]. Pharmacological hyper-activation of this pathway could be achieved by inhibition of PTEN.

It remains open to what extent these findings will have clinical implications. *In vitro*, overexpression and/or amplification have been shown to represent important mechanisms of secondary resistance to imatinib [48]. *In vivo*, however, amplification of *Bcr-Abl* has rarely been observed to be causal for clinical resistance to imatinib. The majority of CML patients with a chronic phase resistant to imatinib develop imatinib resistant cell clones harboring point mutations in the kinase domain of *Bcr-Abl*. Interestingly, the major risk factor for developing resistance during chronic phase is poor compliance [49]. From this it could be concluded that these inadvertent interruptions of imatinib treatment predispose for resistance caused by *Bcr-Abl* point mutations but not for clones harboring an amplified *Bcr-Abl* gene. This clinical observation is well in accordance with our results. Nevertheless, as interruptions of imatinib treatment favor the development of resistant *Bcr-Abl* mutants, therapy free intervals cannot be recommended for clinical study.

A second clinical observation also supports the relevance of our experiments. In a few CML patients multiple copies of the *Bcr-Abl* oncogene have been observed after transformation to blast crisis [50]. Interestingly, in one of these patients the cell clone with *Bcr-Abl* amplification completely disappeared after discontinuation of imatinib [50]. Again, our *in vitro* finding that imatinib withdrawal induces cell death in Bcr-Abl over-expressing cell clones provides a possible mechanism for this phenomenon.

Imatinib monotherapy in previously treated patients with Ph-positive ALL produced a response which was only modest and short-lived [51]. Therefore, these patients are currently treated with the combination of imatinib, chemotherapeutics, and corticosteroids [52], [53]. *In vitro* data from us and others indicate a complex interaction between Bcr-Abl activity and effects of corticosteroids. Bcr-Abl protects cells from corticosteroid-induced cell death [54], [55]. Concomitantly, corticosteroids render pre-B cells more permissive for excessive activity of Bcr-Abl as supported by our observation that the transforming capacity of Bcr-Abl is increased in presence of prednisolone or betamethasone. Moreover, imatinib resistant Bcr-Abl over-expressing cells are rescued from imatinib withdrawal induced cell death when cultured in presence of corticosteroids. The molecular mechanism for this protection from oncogenic stress is the reversal of Bcr-Abl induced changes in cellular metabolism. Indeed, corticosteroids have been shown to reduce glycolysis in macrophages and thymocytes [56], [57]. From these data it may be hypothesized that corticosteroids provide survival advantages for tumor cells upon oncogenic stress and contribute to the pathogenesis of malignant disease. Indeed, clinical observations demonstrated that corticosteroid treatment may support the development of malignant tumors. Corticosteroids have been shown to increase skin cancer risk in non-Hodgkin's lymphoma [58] and enhance risk of bladder cancer [59]. From our finding that corticosteroids are capable of antagonizing the cell death induced by imatinib withdrawal *in vitro*, it may be hypothesized, that intermittent treatment with imatinib combined with continuous steroid therapy would be hazardous. The schedules used for treatment of ALL, however, usually combine continuous treatment of imatinib with intermittent steroid therapy. These schedules should not facilitate resistance development according to our data. This also might explain why amplification as a resistance mechanism is very rare in Bcr-Abl positive ALL. To our knowledge there is no such case described in the literature.

Together, these observations demonstrate that unexpected and complex interactions may occur when antitumor drugs with different mechanisms of action are combined for treatment of malignant disease. To further complicate the issue, these interactions are in part schedule dependent. Preclinical studies using other cell models and primary cells are required to better understand potential risks of combining different drugs. In addition, careful clinical observation aware of these risks is of utmost importance to timely detect potential harmful effects of such combinations.

## Materials and Methods

### Cell culture

The BaF3 was provided by the German Collection of Microorganisms and Cell Cultures (DSMZ, Germany). BaF3p190 was obtained by transfecting the murine factor dependent pro-B cell line BaF3 with pSRαMSVtkneo-p190e1a2. Cells were cultured in RPMI 1640 medium (Gibco), supplemented with 10% heat inactivated fetal bovine serum, 50 µg/ml streptomycin and penicillin (Gibco), 20 mM L-glutamine (or in concentrations as indicated) and maintained in a humidified 95% air-5% CO2 atmosphere at 37°C. Resistant clones were achieved as described previously [60] by cultivating Bcr-Abl positive cells in semisolid medium in the presence of 2 µM imatinib. Cell clones were characterized for kinase domain mutations.

### Reagents

Imatinib mesylate was provided by Research Chemicals inc. Imatinib was used at a concentration of 2 µM. The Prestwick Chemical Library® containing 1,200 small molecules was obtained from Prestwick-Chemical and used at a concentration of 2 µg/ml. 2-deoxy-D-glucose (Sigma, Germany) was used at 1 mM. 6-diazo-5-oxo-l-norleucine (DON; Sigma, Germany) was used at 1 µM. 3-Methyladenin (Sigma, Germany) was used at a concentration of 300 µM. ABT-737 (Toronto Research Chemicals, Canada) was used at 1 µM. P38 MAPK Inhibitor III (Calbiochem, USA) was used at 2 µM. Necrostatin-1 (Sigma, Germany) were used at 50 µM. Prednisolone and betamethasone (Sigma, Germany) were used at a concentration of 2 µg/ml. The pan-caspase inhibitor zVADfmk (Calbiochem, USA) was used at 50 µM.

### Analysis of protein expression

The cellular pellet was resuspended in Laemmli buffer, boiled for 5 min at 97°C and sonicated as described [61]. Following electrophoresis proteins were transferred to nitrocellulose membranes (Schleicher & Schuell Bioscience, Germany). The blots were blocked in 5% nonfat milk in TBS-Tween and incubated with the primary antibodies: anti-Bcl-xL (BD Pharmingen, USA), anti-Abl, anti-p-Tyr-100, anti-(Tyr207)Crkl, anti-(ser473)AKT, anti-AKT, anti-(Tyr202/Tyr204)p44/42, anti-(Tyr694)-Stat5, anti-(Thr180/Tyr182)p38, anti-p38, anti-(Ser51)eIF2α, anti-CHOP, anti-Bim, anti-Beclin-1, anti-ATG7 (Cell Signaling Technologies, USA), and anti-β-actin (Sigma, Germany). Afterwards, blots were incubated with secondary antibody conjugated to horseradish peroxidase (Cell Signaling Technologies, USA) and signals were detected by chemiluminescence (Pierce Biotechnology, USA).

### Detection of cell death

Induction of cell death was assessed by FITC-conjugated Annexin V (Pharmingen, USA) and propidium iodide (PI; Sigma, Germany) staining. The staining was performed according to manufacturers' instructions and analyzed by flow cytometry.

### Cell cycle analyses

Cells were pulse-treated with 10 µM BrdU for 45 min, then pelleted and fixed in ethanol. Cells were stained with propidium iodide (PI) and analysis was performed by determination of DNA content using flow cytometry (Becton Dickinson, Germany).

### Growth inhibition analysis

Growth inhibition and IC_50_ was assessed from the changes in mitochondrial activity after 48 hours of imatinib treatment using MTT assay [62].

### siRNA experiments

For silencing we used siGenome SMARTpool siRNA (Dharmacon, UK). Transfection was performed as previously described [63]. In brief, cells were set to a density of 3.2×10^6^/ml. 800 µl of this cell suspension were mixed with 650 µmol siRNA in a 4 mm electroporation cuvette (Peqlab, Germany) and electroporated. Sequences targeted by SMARTpool siRNAs are listed in table 1. As a control we used Non-Targeting siRNA#1 (Dharmacon, UK). 16 hours after transfection imatinib was washed out. 24 hours after imatinib withdrawal transfection was repeated. After 48 h cell death was quantified. The efficacy of siRNA silencing was tested by Western Blot.

**Table 1:** siRNA target sequences.

gene	siRNA1	siRNA2	siRNA3	siRNA4
**BIM**	UUACAACUGUUACGCUUUA	GGAGACGAGUUCAACGAAA	UGAUGUAAGUUCUGAGUGU	GGGUGUUUGCAAAUGAUUA
**CHOP**	GGAAGCAACGCAUGAAGGA	GAGCAAGGAAGAACUAGGA	CAACAGAGGUCACACGCAC	GGUAUGAGGAUCUGCAGGA
**Beclin-1**	GGAAGAGGCUAACUCAGGA	GGAGUGGAAUGAAAUCAAU	GGGAGUAUAGUGAGUUUAA	GUACCGACUUGUUCCCUAU
**ATG7**	GAUACAAGCUUGGCUGCUA	GCUAGAGACGUGACACAUA	GGCAGCCUCUGUAUGAAUU	GGUCGUGUCUGUCAAGUGC

### Transfection experiments

BaF3 cells were transfected with the retroviral vector pSRαMSVtkneo-*p190e1a2* by electroporation using a double-pulse protocol (first pulse: 750 V, 25 µF, 99 Ω, second pulse: 120 V, 1,500 µF, 99 Ω). Transfected cells were transferred to medium containing 1 ng/ml mIL-3 and 2 µg/ml betamethasone or prednisolone. Control cells were transferred to medium without corticosteroids. After two days, mIL-3 was deprived and 1×10^6^ cells were further cultivated in semi-solid medium. The semi-solid layer was covered by another layer of medium with or without betamethasone or prednislone. After one week colonies were counted and 10 clones each were picked and analyzed for Bcr-Abl expression and autophosphorylation.

### Microscopy

For analysis of the vacuolization cells were incubated with 50 µM ER-Tracker™Green dye (Invitrogen, Germany) and analyzed by conventional fluorescence microscopy (Leica, Germany).

### Metabolomics

The concentrations of glucose, lactate, pyruvate, fumarate, malate, α-ketoglutarate, cis-aconitate, isocitrate, citrate, and proteinogenic amino acids were determined by GC-MS as described [25], [26].

Glucose-6-phosphate, fructose-6-phosphate, phosphoenolpyruvate, 3-phosphoglycerate, fructose-1,6-bisphosphate, 6-phosphogluconate, sedoheptulose-7-phosphate, ribose-5-phosphate, ribulose-5-phosphate, and glucose-1-phosphate were determined by LC-MS-MS [27] using [^13^C_6_]glucose-6-phosphate, [^13^C_6_]glucose-1-phosphate, [^13^C_3_]phosphoenolpyruvate, and [^13^C_6_]fructose-1,6-bisphosphate as internal standards.

### XBP-1 RT-PCR splicing assay

The detection of XBP-1 splicing variants was performed as described by [64].

### Quantification of ATP and protein content

Quantification of ATP was performed using the ATP tumor chemosensitivity assay (DCS Diagnostics, Germany) with 16,000 cells/well in triplicates according to the manufacturers' instructions.

For quantification of total cellular protein content 1×10^6^ cells were collected by centrifugation and dissolved in lysis buffer (50 mM Tris-HCl, pH 7.6; 250 mM NaCl; 0.1% Triton X-100; 5 mM EDTA; 1 µg/ml leupeptin; 1 mM phenylmethylsulfonyl fluoride (PMSF); 1 mM dithiothreitol (DTT); 1 µg/ml aprotinin). Protein content of the extracts was then determined using Advanced Protein Assay (Cytoskeleton Inc, USA) according to the manufacturers' instructions with bovine serum albumin as a protein standard.

### Statistics

Data are expressed as standard deviation of the means (SD). Changes in paired samples were analyzed using two-sided paired *t*-Test.

## Supporting Information

Figure S1 **Analysis of phosphatidyl serine (quantified by Annexin V FITC) versus cell permeability (quantified by propidium iodide) by flow fluorocytometry in BaF3p190 IMR cells incubated with or without prototypical inducers of apoptosis and necrosis.** Cells were incubated with/without staurosporine as apoptotic control or H_2_O_2_ as necrotic control (Zhang *et al.*, 2009*) for 4 hours and then stained with Annexin V or propidium iodide alone (upper two panels) or with the combination of both Annexin V and propidium iodide (lower panel). *Zhang H, Zhong C, Shi L, Guo Y, Fan Z. (2009). Granulysin Induces Cathepsin B Release from Lysosomes of Target Tumor Cells to Attack Mitochondria through Processing of Bid Leading to Necroptosis. J Immunol 182: 6993–7000.
(TIF)

Figure S2 **The second generation Bcr-Abl inhibitors dasatinib and nilotinib reduce Bcr-Abl activity and rescue Bcr-Abl over-expressing cell clones from imatinib withdrawal induced cell death.** Left panel: Bcr-Abl protein level and autophosphorylation in imatinib-sensitive cells (p190wt) in comparison to imatinib-resistant cell clones (IMR6 and IMR10) in the presence or absence of 2 µM imatinib, 100 nM dasatinib, or 75 µM nilotinib. Right panel: induction of cell death in cells cultivated in presence or absence of imatinib, dasatinib, or nilotinib.
(TIF)

Figure S3 **Cellular ATP levels in imatinib deprived cells incubated with or without 2-DG or DON.** Cells were cultivated in presence or absence of 1 mM 2-DG or 1 µM DON for 48 hours and then harvested for ATP quantification. Values are presented as relative to controls (cells cultivated with imatinib) and reflect means ± SD from triplicates.
(TIF)

Figure S4 **Inhibition of autophagy has no effect on induction of cell death upon Bcr-Abl hyper-activation.** (A) 3-MA has no effect on imatinib withdrawal induced cell death. Cells were pre-treated with the authophagy inhibitor 3-MA prior to Imatinib withdrawal. After 48 hours cells were harvested for cell death quantification by Annexin V staining and flow cytometry. (B) Down-modulation of central regulators of autophagy has no influence on imatinib withdrawal induced cell death. Cells were transfected with control siRNA or siRNA targeting Beclin (left panel) or ATG7 (right panel), cultivated with/without Imatinib for 48 h, and then harvested and analyzed for protein levels and cell death.
(TIF)

Figure S5 **Inhibition of Caspase activity by zVADfmk enhances, rather than blocks cell death development.** Cells were pre-treated with 50 µM zVAD-fmk for 2 hours before imatinib withdrawal. After 48 h cells were harvested and cell death was quantified by Annexin V-staining.
(TIF)

Figure S6 **Characterization of early cell death after imatinib withdrawal in cells pretreated with ABT-737 by Annexin V and propidium iodide staining.** Cells were cultivated in the presence and absence of imatinib and ABT-737 for 24 hours and then harvested for Annexin V or propidium iodide single staining.
(TIF)
