# Supplementary figures and images for: Oncogenic Stress Induced by Acute Hyper-Activation of Bcr-Abl Leads to Cell Death upon Induction of Excessive Aerobic Glycolysis
Source: PLoS One. 2011 Sep 20;6(9):e25139. doi: 10.1371/journal.pone.0025139 (PMC3176818; doi:10.1371/journal.pone.0025139)

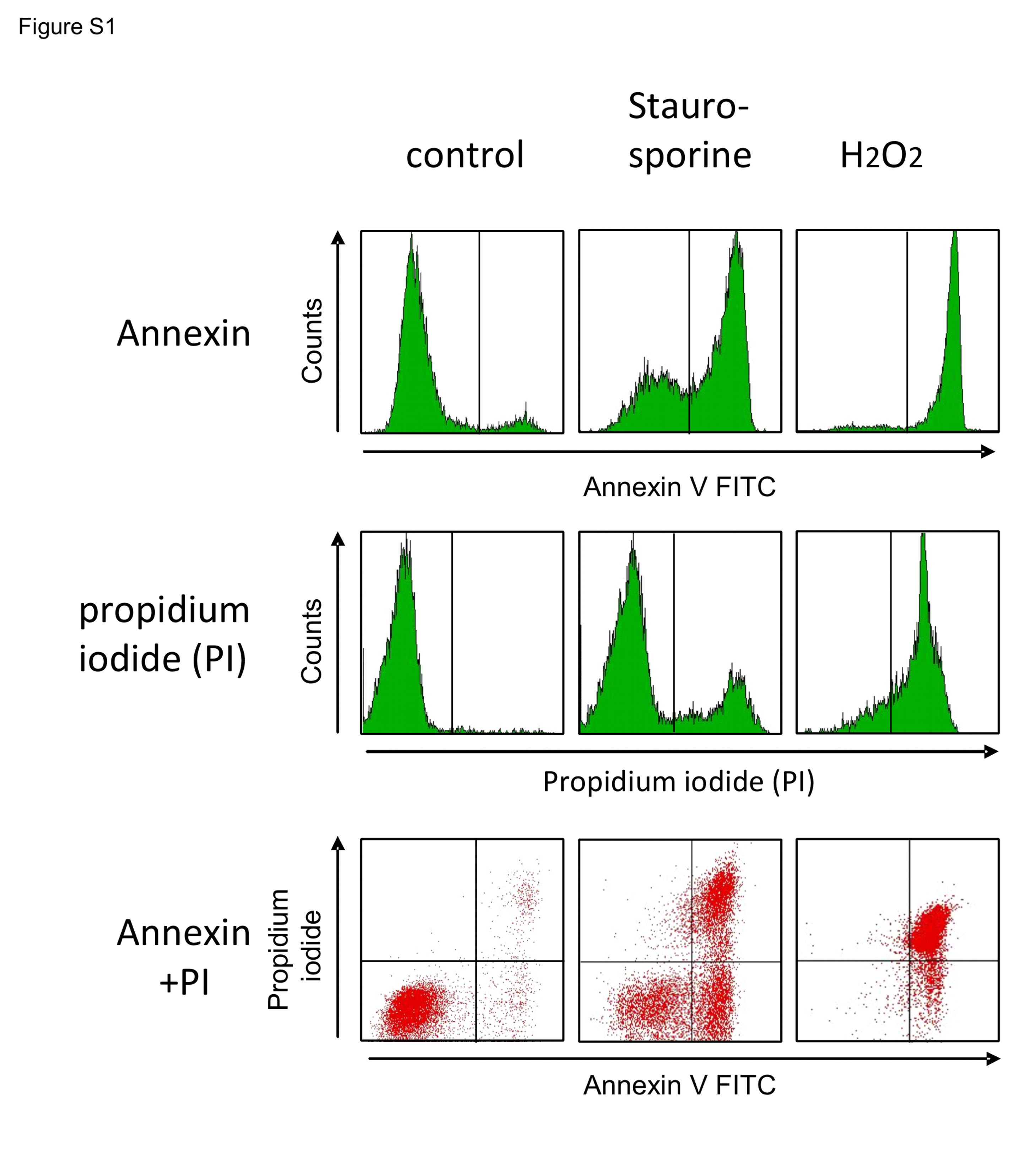

Supplement: Figure S1 — Analysis of phosphatidyl serine (quantified by Annexin V FITC) versus cell permeability (quantified by propidium iodide) by flow fluorocytometry in BaF3p190 IMR cells incubated with or without prototypical inducers of apoptosis and necrosis. Cells were incubated with/without staurosporine as apoptotic control or H2O2 as necrotic control (Zhang et al., 2009*) for 4 hours and then stained with Annexin V or propidium iodide alone (upper two panels) or with the combination of both Annexin V and propidium iodide (lower panel). *Zhang H, Zhong C, Shi L, Guo Y, Fan Z. (2009). Granulysin Induces Cathepsin B Release from Lysosomes of Target Tumor Cells to Attack Mitochondria through Processing of Bid Leading to Necroptosis. J Immunol 182: 6993–7000. (TIF) [file pone.0025139.s001.tif]

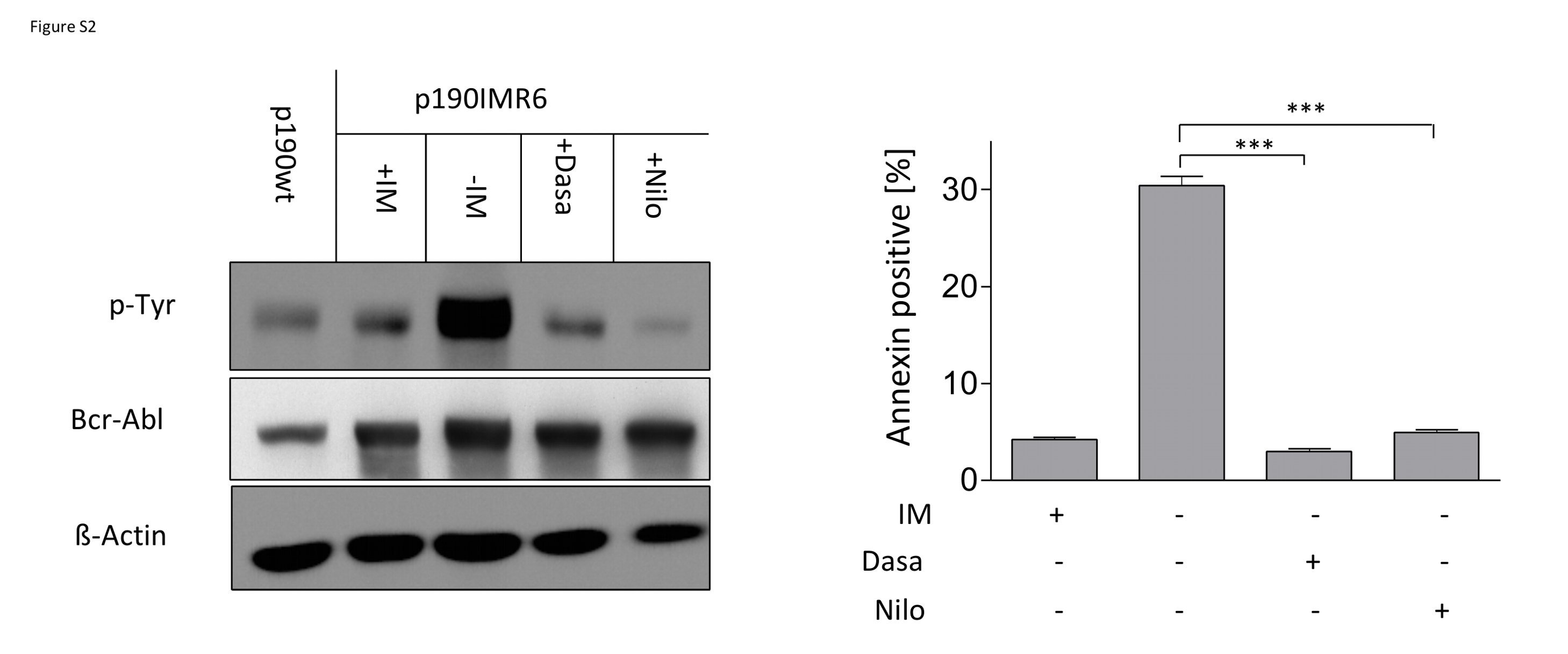

Supplement: Figure S2 — The second generation Bcr-Abl inhibitors dasatinib and nilotinib reduce Bcr-Abl activity and rescue Bcr-Abl over-expressing cell clones from imatinib withdrawal induced cell death. Left panel: Bcr-Abl protein level and autophosphorylation in imatinib-sensitive cells (p190wt) in comparison to imatinib-resistant cell clones (IMR6 and IMR10) in the presence or absence of 2 µM imatinib, 100 nM dasatinib, or 75 µM nilotinib. Right panel: induction of cell death in cells cultivated in presence or absence of imatinib, dasatinib, or nilotinib. (TIF) [file pone.0025139.s002.tif]

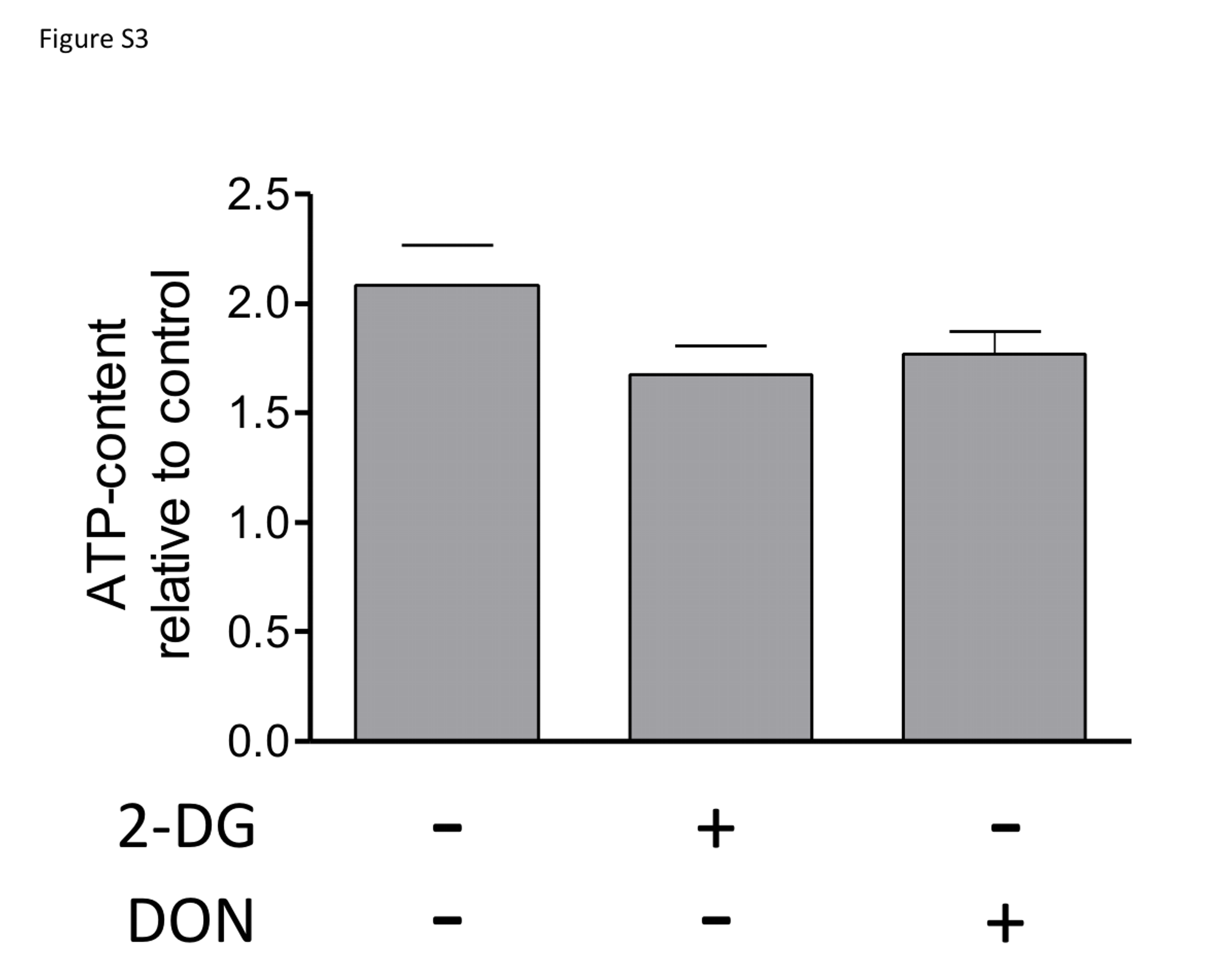

Supplement: Figure S3 — Cellular ATP levels in imatinib deprived cells incubated with or without 2-DG or DON. Cells were cultivated in presence or absence of 1 mM 2-DG or 1 µM DON for 48 hours and then harvested for ATP quantification. Values are presented as relative to controls (cells cultivated with imatinib) and reflect means ± SD from triplicates. (TIF) [file pone.0025139.s003.tif]

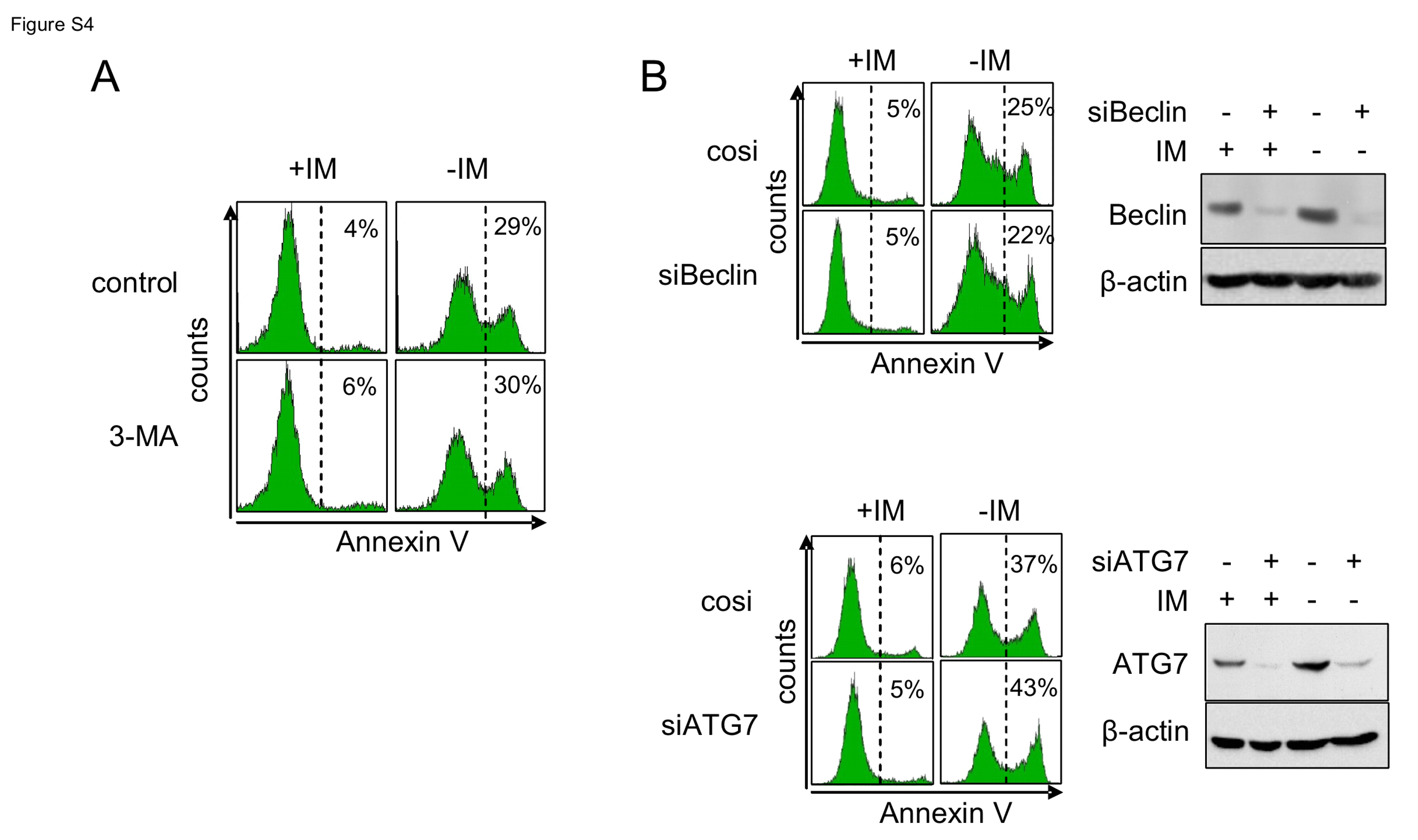

Supplement: Figure S4 — Inhibition of autophagy has no effect on induction of cell death upon Bcr-Abl hyper-activation. (A) 3-MA has no effect on imatinib withdrawal induced cell death. Cells were pre-treated with the authophagy inhibitor 3-MA prior to Imatinib withdrawal. After 48 hours cells were harvested for cell death quantification by Annexin V staining and flow cytometry. (B) Down-modulation of central regulators of autophagy has no influence on imatinib withdrawal induced cell death. Cells were transfected with control siRNA or siRNA targeting Beclin (left panel) or ATG7 (right panel), cultivated with/without Imatinib for 48 h, and then harvested and analyzed for protein levels and cell death. (TIF) [file pone.0025139.s004.tif]

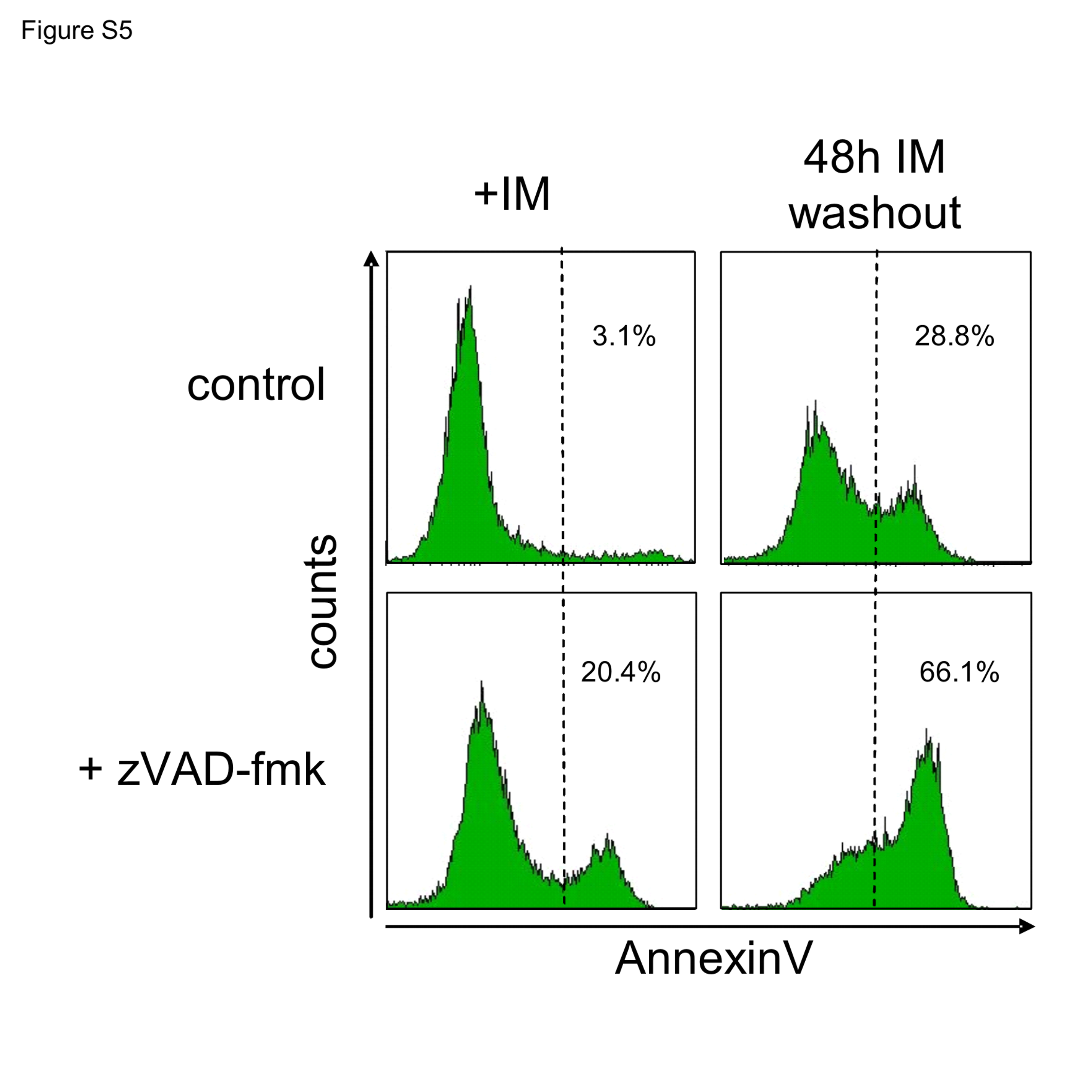

Supplement: Figure S5 — Inhibition of Caspase activity by zVADfmk enhances, rather than blocks cell death development. Cells were pre-treated with 50 µM zVAD-fmk for 2 hours before imatinib withdrawal. After 48 h cells were harvested and cell death was quantified by Annexin V-staining. (TIF) [file pone.0025139.s005.tif]

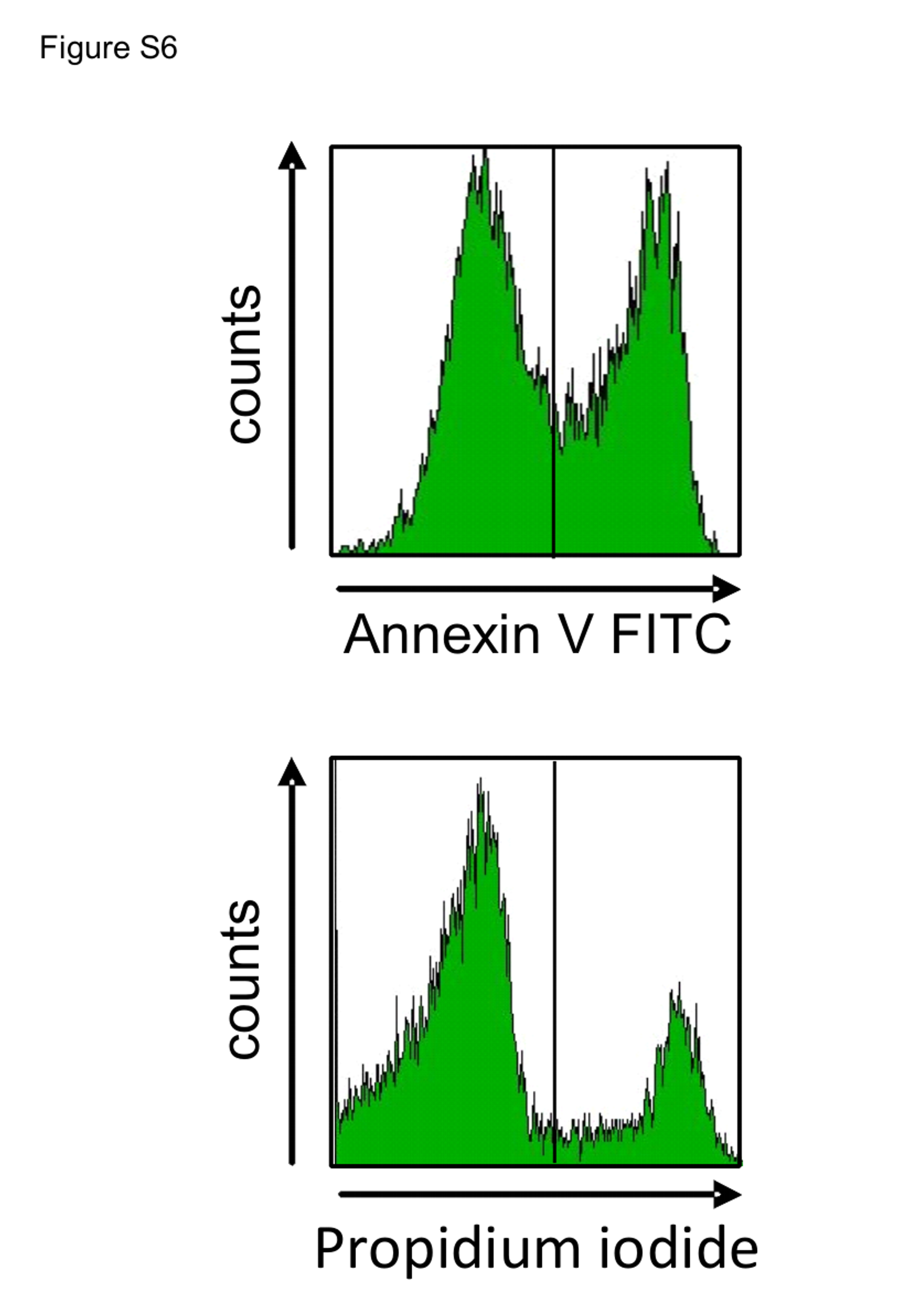

Supplement: Figure S6 — Characterization of early cell death after imatinib withdrawal in cells pretreated with ABT-737 by Annexin V and propidium iodide staining. Cells were cultivated in the presence and absence of imatinib and ABT-737 for 24 hours and then harvested for Annexin V or propidium iodide single staining. (TIF) [file pone.0025139.s006.tif]
